# scMOG: A graph neural network method for regulatory relationship‐preserving single‐cell multi‐omics integration

**DOI:** 10.1002/qub2.70041

**Published:** 2026-05-06

**Authors:** Yucheng Lu, Xun Zhang, Hongwei Li

**Affiliations:** ^1^ School of Mathematics and Physics China University of Geosciences Wuhan China

**Keywords:** contrastive learning module, heterogeneous graph neural network, integration, omics alignment module, single‐cell multi‐omics

## Abstract

Single‐cell multi‐omics sequencing technology provides a powerful tool for studying cellular heterogeneity. However, beyond the challenges of sparsity, heterogeneity, and dimensionality differences, a critical challenge in multi‐omics data integration lies in preserving the true regulatory relationships among molecular features. To address these limitations, we propose single‐cell multi‐omics graph neural networks (scMOG), a framework that leverages heterogeneous graphs to preserve regulatory relationships in single‐cell multi‐omics data. scMOG leverages encoders to extract low‐dimensional embeddings of both cells and features while reconstructing the input data using zero‐inflated negative binomial decoders, effectively handling high sparsity and noise. In addition, scMOG introduces a contrastive learning module and an omics alignment module to preserve differences in expression patterns across distinct omics while extracting consistent information. Experimental results on eight single‐cell multi‐omics datasets demonstrate that scMOG outperforms existing methods, producing embeddings that capture meaningful biological signals. scMOG provides an effective solution for integrating single‐cell multi‐omics data, offering a scalable framework that preserves regulatory signals.

## 1 INTRODUCTION

The emergence of single‐cell RNA sequencing (scRNA‐seq) technology has provided a comprehensive cellular landscape at the single‐cell level, enhancing our understanding of cellular heterogeneity [1, 2, 3]. In recent years, advancements in sequencing technologies have led to the generation of multiple types of omics data, including the transcriptome, genome, epigenome, proteome, and metabolome. Current sequencing technologies have enabled the simultaneous measurement of multiple omics layers [4]. For example, single‐cell assay of transposase‐accessible chromatin sequencing (scATAC‐seq) can detect chromatin accessibility, whereas single‐cell chromatin accessibility and transcriptome sequencing (scCAT‐seq) allows the simultaneous analysis of the transcriptome and epigenome [5]. Similarly, the cellular index of transcriptomes and epitopes by sequencing (CITE‐seq) enables the simultaneous analysis of the transcriptome and cell surface protein expression levels [6].

scRNA‐seq data are characterized by high dimensionality, high sparsity, and high noise levels [7]. To address these challenges, numerous algorithms have been developed for their analysis [8, 9]. Prior to downstream analyses, scRNA‐seq data typically require clustering [10]. However, examining single‐cell data from a single perspective may fail to capture a comprehensive view of cellular states. Integrating multiple omics layers can provide complementary insights [11, 12, 13]. For instance, proteomic data, which capture proteins bound to antibodies during measurement, tend to be less sparse, making them useful for identifying common cell types. In contrast to scRNA‐seq data, scATAC‐seq data contain more cell type‐specific information, as such information is primarily located in enhancer regions, which are effectively captured by scATAC‐seq technology [14]. Although clustering based on a single omics layer remains useful, it may overlook broader regulatory interactions and cross‐omics dependencies within cells. Therefore, integrating multiple omics layers can offer a more comprehensive approach to cell identification [15].

With the development of sequencing technologies, various multi‐omics integration methods have emerged. Traditional single‐cell multi‐omics integration approaches primarily fall into two categories: matrix factorization‐based and manifold alignment‐based methods. Matrix factorization methods, such as multi‐omics factor analysis (MOFA) [16], employ a probabilistic Bayesian framework to jointly decompose multi‐omics data through shared factor matrices, extending principal component analysis (PCA) to multi‐omics applications while maintaining interpretability. Coupled NMF [17] applies non‐negative matrix factorization to single‐cell genomic data, projecting two omics matrices into low‐dimensional spaces and introducing a coupling matrix that leverages gene regulatory relationships to establish cross‐omics linear mappings, thereby enhancing feature consistency. However, such linear approaches often struggle to capture complex nonlinear cross‐omics relationships. In contrast, manifold alignment methods such as MACHER [18] utilize Gaussian process latent variable models to infer shared pseudotemporal trajectories across omics modalities, revealing coordinated transcriptomic and epigenomic dynamics. Although these nonlinear methods better model biological complexity, they typically require stronger prior assumptions and involve higher computational costs.

Deep learning has facilitated progress in a variety of computational tasks across different omics [19, 20, 21]. Variational autoencoders (VAE) [22] have been demonstrated to be effective in scRNA‐seq batch effect removal [23] and have also been widely adopted for multi‐omics data integration. For instance, totalVI [24] integrates paired transcriptomic and proteomic data using VAE, whereas Cobolt [25] assumes shared latent variables across omics modalities to capture biological signals, employing VAE to infer latent representations as cross‐omics cell embeddings. DCCA [26] combines VAE with attention transfer to jointly model transcriptomic and epigenomic data from the same cells. Unlike DCCA’s attention transfer, scMIC [27] enhances latent embedding similarity through multiple collaborative supervised clustering modules. However, these methods often neglect regulatory relationships between omics features and fail to account for the varying importance of different omics, despite transcriptomic data’s richer information content [28]. scDRMAE [29] employs parallel masked autoencoders with ResNet‐like architectures [30] to preserve RNA‐seq information during fusion but overlooks cross‐feature interactions. SIMBA [31] is a versatile single‐cell embedding framework that embeds both cells and features into a shared latent space, enabling unified downstream analysis. A critical challenge remains in addressing the high sparsity of gene expression and chromatin accessibility data through appropriate data distribution models. In addition, existing methods predominantly focus on learning cell‐level embeddings while neglecting the explicit extraction of feature embeddings, which poses challenges for downstream analysis.

Inspired by prior work on scRNA‐seq data dimensionality reduction [32] and integration [33], we propose scMOG, a single‐cell multi‐omics integration model specifically designed for paired data that integrates two modalities measured in the same cells simultaneously. For each omics layer, scMOG employs parallel graph autoencoder architecture. Leveraging feature expression levels across cells and prior feature relationships, the model utilizes graph neural network (GNN)‐based encoders to generate high‐quality representations that effectively fuse feature dependencies. An omics alignment loss connects the parallel graph autoencoders to align low‐dimensional embeddings of identical cells while eliminating redundant information. To address high noise and sparsity in single‐cell data, scMOG estimates modality‐specific distributions to prevent information loss during encoding. Additionally, a contrastive learning module is integrated into the shared space for each modality to enhance discriminative cell embeddings. The modules of scMOG work synergistically to learn cell representations in an unsupervised manner. We evaluated scMOG on real single‐cell multi‐omics datasets and compared it with nine benchmark methods. Extensive experimental results demonstrate that scMOG outperforms benchmark methods.

## 2 RESULTS

This section first provides an overview of scMOG. To evaluate the performance of scMOG in integrating paired single‐cell multi‐omics datasets, experiments were conducted on datasets from different species and tissues. Detailed information on all datasets used can be found in Table 1. Comparisons were made with nine benchmark algorithms to validate the robustness and scalability of scMOG in integrating single‐cell multi‐omics data. The evaluation metrics included adjusted Rand index (ARI) [34], normalized mutual information (NMI) [35], and accuracy (ACC) [36], along with clustering result visualization [37]. Furthermore, the relationships between features in the shared space are evaluated using mean squared error (MSE) and accuracy values.

**TABLE 1:** Quantitative information of all datasets.

Dataset	No. of cells	No. of genes	No. of peaks/proteins	Species	Tissue/organ	Technology
Ma‐1	5692	21,478	340,341	Mouse	Skin	SHARE‐seq
Ma‐2	5927	21,478	340,341	Mouse	Skin	SHARE‐seq
PBMC‐1	11,909	36,601	108,377	Human	PBMC	10x Multiome
PBMC‐2	10,137	36,601	139,620	Human	PBMC	10x Multiome
Chen	9190	28,930	241,757	Mouse	Cortex	SNARE‐seq
Spector	3762	33,538	49	Human	PBMC	10x Multiome
SLN208‐1	8715	13,553	209	Mouse	Spleen and lymph node	CITE‐seq
SLN208‐2	7105	13,553	209	Mouse	Spleen and lymph node	CITE‐seq

### 2.1 The overview of scMOG

Suppose there are M omics layers, each represented by a matrix $X_{i}{\in R}^{n\times l_{i}}$, where n denotes the number of cells and $l_{i}$ represents the feature dimension of the i‐th omics. The goal of deep learning‐based integration methods is to find a mapping function F that projects multi‐omics data into a unified latent space, which can be mathematically formulated as follows:

$$F:\left\{X_{1},X_{2},\text{…},X_{M}\right\}\mapsto Z{\in R}^{n\times d},$$

where Z is the low‐dimensional joint representation of cells, and d is the dimension of the latent space.

The workflow of scMOG is illustrated in Figure 1. This method is based on a graph autoencoder framework, with the primary goal of obtaining effective low‐dimensional embeddings of cells and features by integrating information from different omics. In the low‐dimensional space, it aims to preserve biological information as much as possible while maintaining the correlations between features. Ultimately, the low‐dimensional embeddings of cells derived from gene expression data are used for clustering and visualization, whereas the feature embeddings facilitate the quantitative analysis of preserved transcriptional regulatory information and protein–protein interaction data.

**FIGURE 1:**
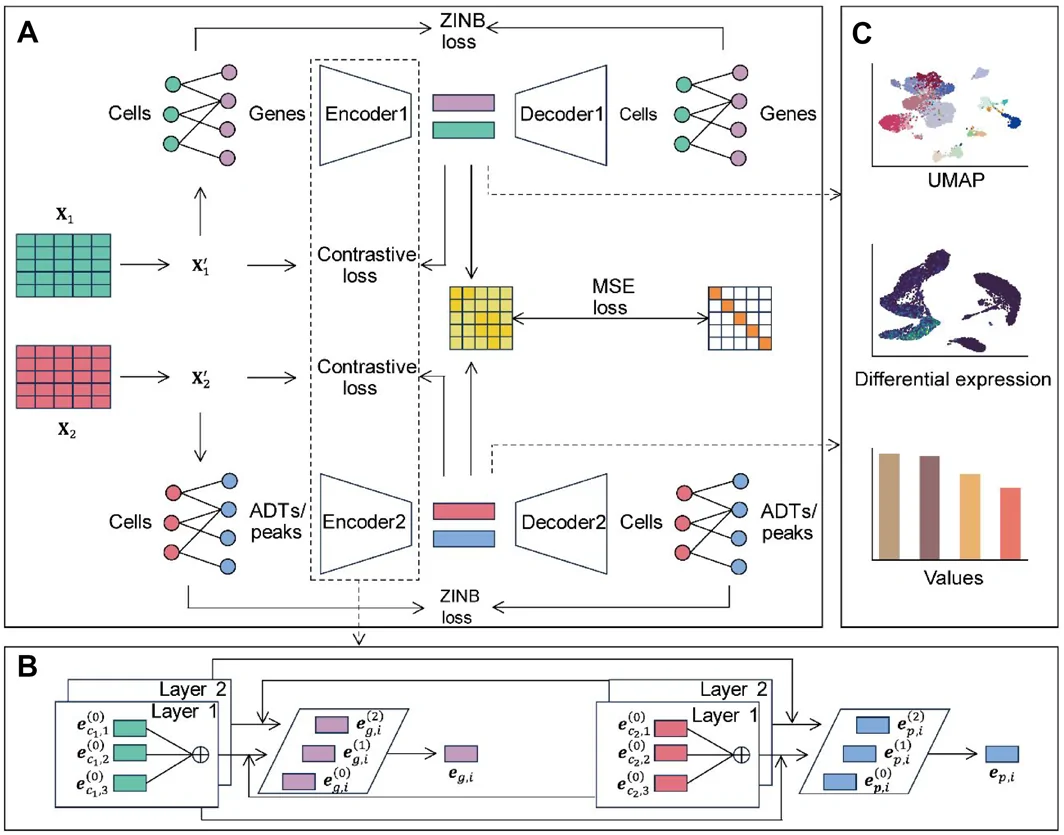
Overview of the scMOG. (A) Input data preprocessing and module design. Given raw transcriptomic data $X_{1}$ and another omics data $X_{2}$ (either epigenomics or proteomics), each matrix undergoes separate preprocessing. The two omics layers interact through three key modules: the graph autoencoder module, the contrastive learning module, and the omics alignment module. These modules enable unsupervised learning of both cell and feature representations for each omics layer. (B) Cross‐omics encoding with LightGCN. The input data is encoded using a LightGCN‐based encoder, where ⊕ represents the information aggregation process. When integrating gene expression and chromatin accessibility data, the feature representation of one omics layer incorporates information from the other omics layer that has a regulatory relationship with it at each encoding layer. (C) Performance evaluation of scMOG. The performance of scMOG is assessed through clustering visualization, identification of differentially expressed genes, and the computation of evaluation metrics. ADT, antibody‐derived tag; UMAP, uniform manifold approximation and projection; ZINB, zero‐inflated negative binomial.

The key components of scMOG include the graph autoencoder, contrastive learning module, and omics alignment module, each playing a crucial role. The encoder in the graph autoencoder encodes cells and features into low‐dimensional embeddings, which are then reconstructed into the original data matrix using a zero‐inflated negative binomial (ZINB) decoder. For each input modality, a contrastive learning module is incorporated to ensure that similar cells are pulled closer in the shared space, whereas different cell types are pushed apart, thereby improving integration performance. Additionally, an omics alignment module is introduced to connect the autoencoders of different omics, ensuring that the same cells remain close in the shared space while removing redundant information across omics. In the following sections, we will illustrate each module of scMOG using gene expression data and chromatin accessibility data as an example.

#### 2.1.1 Graph autoencoder

scMOG takes preprocessed gene expression data $X_{1}^{\prime }=\left[x_{ij}^{1}\right]{\in R}^{n\times m_{1}}$ and chromatin accessibility data $X_{2}^{\prime }=\left[x_{ij}^{2}\right]{\in R}^{n\times m_{2}}$ as input, where n is the number of preprocessed cells, whereas $m_{1}$ and $m_{2}$ represent the counts of preprocessed genes and chromatin accessibility regions, respectively. The encoder, based on LightGCN [38], generates low‐dimensional representations for cells: $E_{c_{1}}{\in R}^{n\times d}$, $E_{c_{2}}{\in R}^{n\times d}$, as well as for features: $E_{f_{1}}{\in R}^{m_{1}\times d}$, $E_{f_{2}}{\in R}^{m_{2}\times d}$, where d is the embedding dimension, which is the same for both cells and features in scMOG. Because the heterogeneous graph connects cell nodes and feature nodes directly, each graph convolution performs message passing across the two node types, ensuring that $E_{f_{i}}$ reflects the neighborhood structure shaped by $E_{c_{i}}$, whereas $E_{c_{i}}$ simultaneously integrates regulatory signals encoded in $E_{f_{i}}$. To reconstruct the original data, the Hadamard product [39] is applied to $E_{c_{1}}$ and $E_{f_{1}}$, as well as $E_{c_{2}}$ and $E_{f_{2}}$, followed by a ZINB‐based decoder, which estimates the distribution parameters of the two omics layers.

#### 2.1.2 Contrastive learning module

Given the widespread application of contrastive learning in single‐cell data analysis, scMOG incorporates a contrastive learning module to optimize the low‐dimensional representations of cells. For each omics dataset, mutual nearest neighbor (MNN) pairs are identified in the preprocessed space. These MNN pairs are treated as positive pairs, whereas for each positive pair, a random cell is selected as a negative sample. A triplet loss is used for model training, and its superior performance in scRNA‐seq data integration has been demonstrated [40].

#### 2.1.3 Omics alignment module

The omics alignment module in scMOG is designed to enhance the consistency of cell representations across different omics layers by maximizing their shared structure. Specifically, cosine similarity is computed between the cell embeddings from both omics. Given that each omics dataset contains n cells, the similarity matrix is compared against an n‐dimensional identity matrix using MSE. This objective encourages embeddings of the same cell to be close in the shared latent space while reducing modality‐specific noise, thereby improving cross‐omics alignment and representation consistency.

### 2.2 Effective integration of transcriptomics and epigenomics

To demonstrate that scMOG can effectively integrate transcriptomic and epigenomic data, we first conducted experiments on multiple real datasets from different species and tissues, covering various biological backgrounds of mice and humans. Among the five datasets used in the experiments, Ma‐1 and Ma‐2 are relatively small‐scale, PBMC‐1 and PBMC‐2 contain more than ten thousand cells and are large‐scale, whereas the Chen dataset is of medium scale. We compared scMOG with eight benchmark methods: scMDC [41], scMIC [27], DCCA [26], Cobolt [25], PCA, scGAE [42], SIMBA [31], and scDRMAE [29]. Because totalVI [24] is not applicable for integrating transcriptomics and epigenomics, it was not included in this comparison.

Figure 2A presents the uniform manifold approximation and projection (UMAP) visualization of cell embeddings obtained by different algorithms on the Chen dataset, which consists of 22 cell types. scMOG generates cell representations ($E_{c_{1}}$ and $E_{f_{1}}$) for both omics by leveraging the LightGCN framework. Our experiments revealed that transcriptomic cell representations retain more informative features; therefore, clustering was performed using transcriptomic embeddings, as shown in Table S2. The visualization indicates that scMOG effectively integrates transcriptomic and epigenomic data. Compared to other methods, scMOG, along with scMDC and DCCA, successfully separates different cell types with a well‐defined distribution. For instance, cell types Clau and E5Tshz2 are better distinguished by scMOG. Notably, scGAE, an algorithm specifically designed for scRNA‐seq data, exhibits limited capability in distinguishing cell types on the Chen dataset, suggesting that incorporating multi‐omics data provides richer biological information. Further performance evaluation in Figure 2B demonstrates that scMOG achieves better performance metrics on the Chen dataset (ARI = 0.71, NMI = 0.73, ACC = 0.81).

**FIGURE 2:**
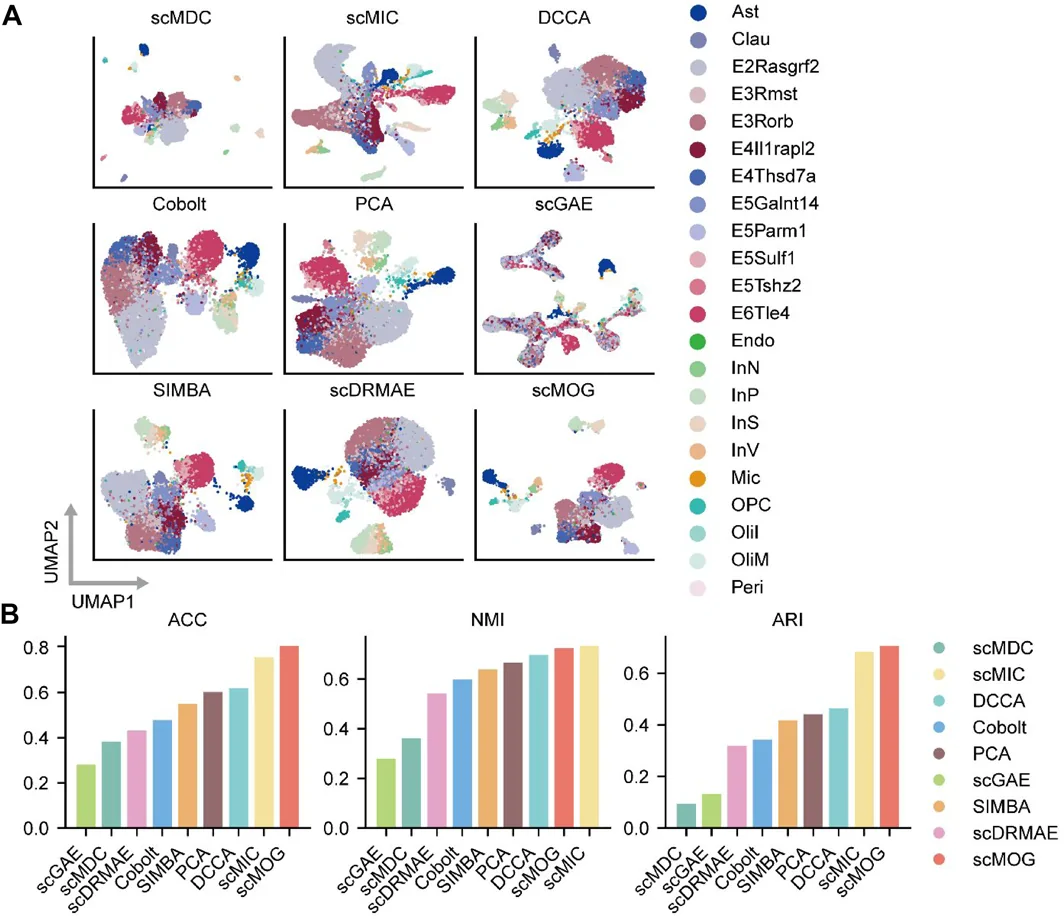
Comprehensive comparison of different algorithms on the Chen dataset. (A) UMAP visualization of cell embeddings obtained by different algorithms, with colors indicating the ground‐truth cell types. (B) Comparison of ACC, NMI, and ARI scores computed by different algorithms on the Chen dataset. ACC, accuracy; ARI, adjusted Rand index; NMI, normalized mutual information; UMAP, uniform manifold approximation and projection.

Besides the Chen dataset, scMOG also demonstrates excellent integration capabilities across the other four transcriptomic and epigenomic datasets (Figures S1–S4). It achieves the highest ARI, NMI, and ACC on the Ma‐1 dataset; the best ARI, second‐best NMI, and ACC on the Ma‐2 dataset; moderate ARI and ACC, but second‐best NMI, on PBMC‐1; and second‐best ARI with moderate ACC and NMI (scMOG = 0.74) on PBMC‐2, where the highest NMI (scMIC = 0.77) is only slightly superior. Additionally, scMOG’s clustering visualizations effectively separate most cells with minimal intercluster mixing (Figures S1–S4), further demonstrating its adaptability and strong performance in various biological scenarios.

In summary, these experimental results confirm that scMOG can effectively integrate transcriptomics and epigenomics. Across multiple datasets, scMOG consistently outperforms benchmark methods in terms of cell type separation and information fusion.

### 2.3 Effective integration of transcriptomics and proteomics

To verify the effectiveness of scMOG in integrating transcriptomic and proteomic data, we conducted experiments on three datasets derived from different species and tissues, including samples from both mice and humans. Because DCCA [26] and Cobolt [25] are not applicable for integrating transcriptomics and proteomics, we compared scMOG with seven other methods: scMDC [41], scMIC [27], totalVI [24], PCA, scGAE [42], SIMBA [31], and scDRMAE [29].

Figure 3A presents the UMAP visualization of cell embeddings obtained using different algorithms on the Spector dataset, which consists of 16 cell types. As shown in the figure, scMOG effectively integrates proteomics and transcriptomics. Compared to benchmark methods, scMOG demonstrates superior cell type separation, revealing a clearer structural distinction between cell populations. Notably, scMOG achieves a more distinct separation between cell type 4 and cell type 5. The scRNA‐seq analysis tool scGAE struggles to effectively distinguish different cell types in the Spector dataset.

**FIGURE 3:**
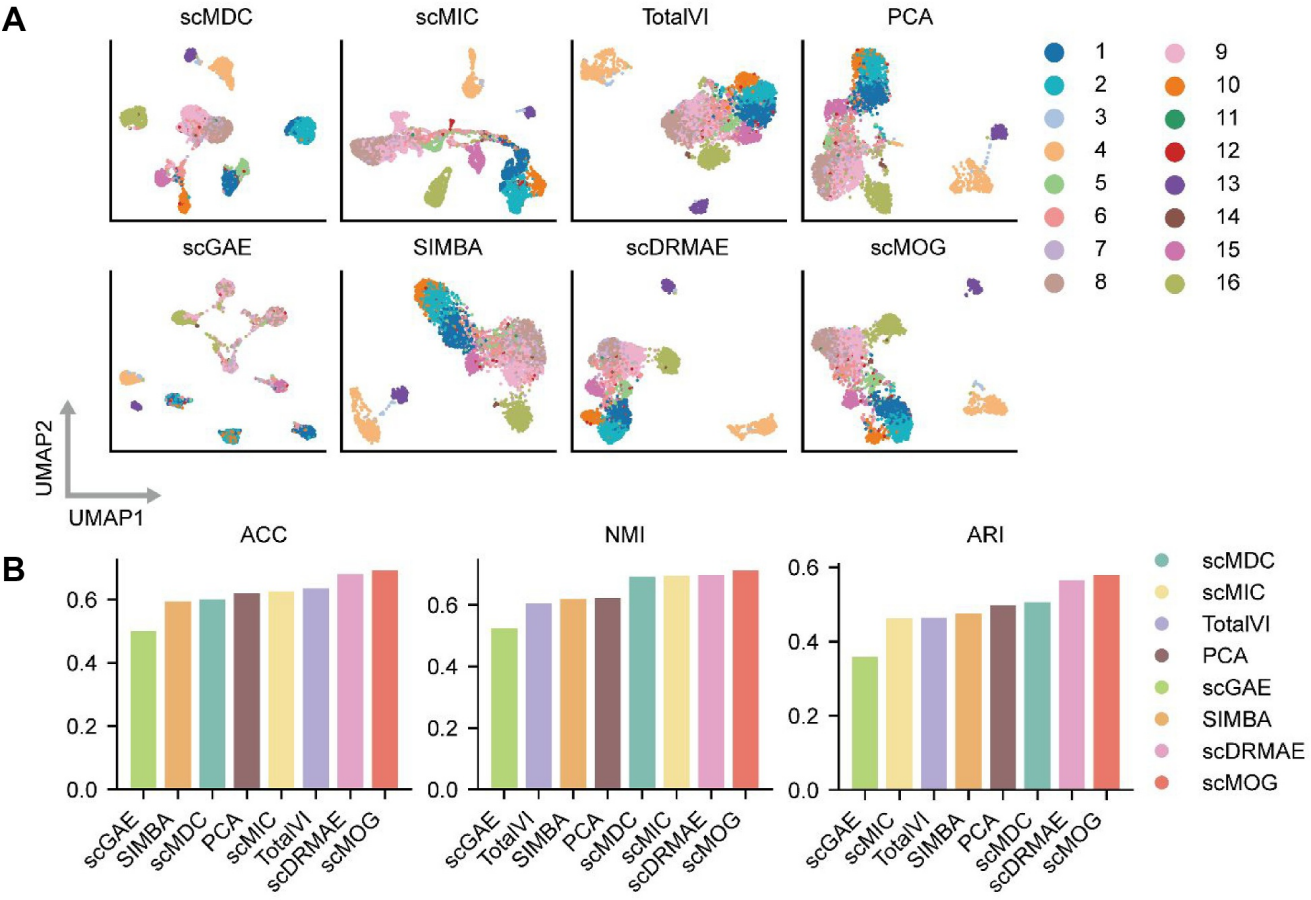
Comprehensive comparison of different algorithms on the human PBMC dataset. (A) UMAP visualization of cell embeddings obtained by different algorithms, with colors indicating the ground‐truth cell types. (B) Comparison of ACC, NMI, and ARI scores computed by different algorithms on the human PBMC dataset. ACC, accuracy; ARI, adjusted Rand index; NMI, normalized mutual information; UMAP, uniform manifold approximation and projection.

The evaluation metrics shown in Figure 3B further highlight the advantages of scMOG. Compared to other methods, scMOG achieves the best performance across all three metrics: ACC (scMOG = 0.69), NMI (scMOG = 0.71), and ARI (scMOG = 0.58), indicating its effectiveness in integrating multi‐omics data. Although scDRMAE achieves similar performance (ACC = 0.68, NMI = 0.70, ARI = 0.56), the gap between scMOG and the third‐ranked method is significantly larger (totalVI‐ACC = 0.64, scMDC/scMIC‐NMI = 0.69, scMDC‐ARI = 0.51).

Beyond the Spector dataset, scMOG also demonstrates strong cross‐omics integration capabilities in two mouse spleen and lymph node datasets. As shown in Figures S5 and S6, scMOG effectively captures cross‐omics information in these datasets, achieving the highest ACC, NMI, and ARI scores. In addition, we provide an omics‐labeled UMAP in Figure S7, both of which show that scMOG produces more intermixed cross‐omics distributions compared with the original embeddings. These results further confirm scMOG’s robust capability in aligning transcriptomic and prote‐omic data.

### 2.4 Preserving regulatory relationships between genes and chromatin regions

Transcriptional enhancers act as distal DNA regulatory elements and play a crucial role in gene expression regulation [43, 44]. scMOG employs encoders based on LightGCN, which not only learn the cell embeddings of both omics ($E_{c_{1}}$ and $E_{c_{2}}$) but also capture the feature embeddings ($E_{f_{1}}$ and $E_{f_{2}}$). We compared scMOG with SIMBA, which also learns embeddings for both cells and features, to evaluate their ability to preserve regulatory relationships between genes and chromatin regions (also referred to as “peaks”). The evaluation of gene–peak associations relies on genomic annotations and the construction of the input graph based on proximity, so the MSE metric mainly reflects the model’s ability to preserve these prior‐informed associations. We preprocessed the gene expression and chromatin accessibility matrices and performed comparisons accordingly. Specifically, PCA50 represents the retention of 50 principal components after PCA preprocessing, whereas PCA64 retains 64 principal components, corresponding to the dimensionalities of low‐dimensional embeddings learned by SIMBA and scMOG, respectively. By computing the gene–peak similarity matrix (see Note S2 and Section 4.2 for details) and comparing it with the gene–peak association matrix derived from genomic annotations, we assessed the performance of different methods in preserving gene–peak correlations. Higher gene–peak similarity values indicate a higher confidence level in the presence of true regulatory relationships.

To quantitatively evaluate similarity, we adopted MSE as the evaluation metric. A lower MSE between the computed gene–peak similarity matrix and the ground‐truth gene–peak association matrix indicates better performance in maintaining true gene–peak relationships. We conducted experiments on five multi‐omics datasets containing epigenomic data, and the MSE results are presented in Figure 4A and Figure S8. The results show that scMOG achieves a lower MSE compared to direct preprocessing. Although its MSE is slightly higher than SIMBA on the PBMC‐1 and PBMC‐2 datasets, scMOG outperforms SIMBA on the other three datasets by achieving lower MSE values. This suggests that scMOG learns biologically meaningful gene and peak embeddings and has an advantage in inferring regulatory relationships.

**FIGURE 4:**
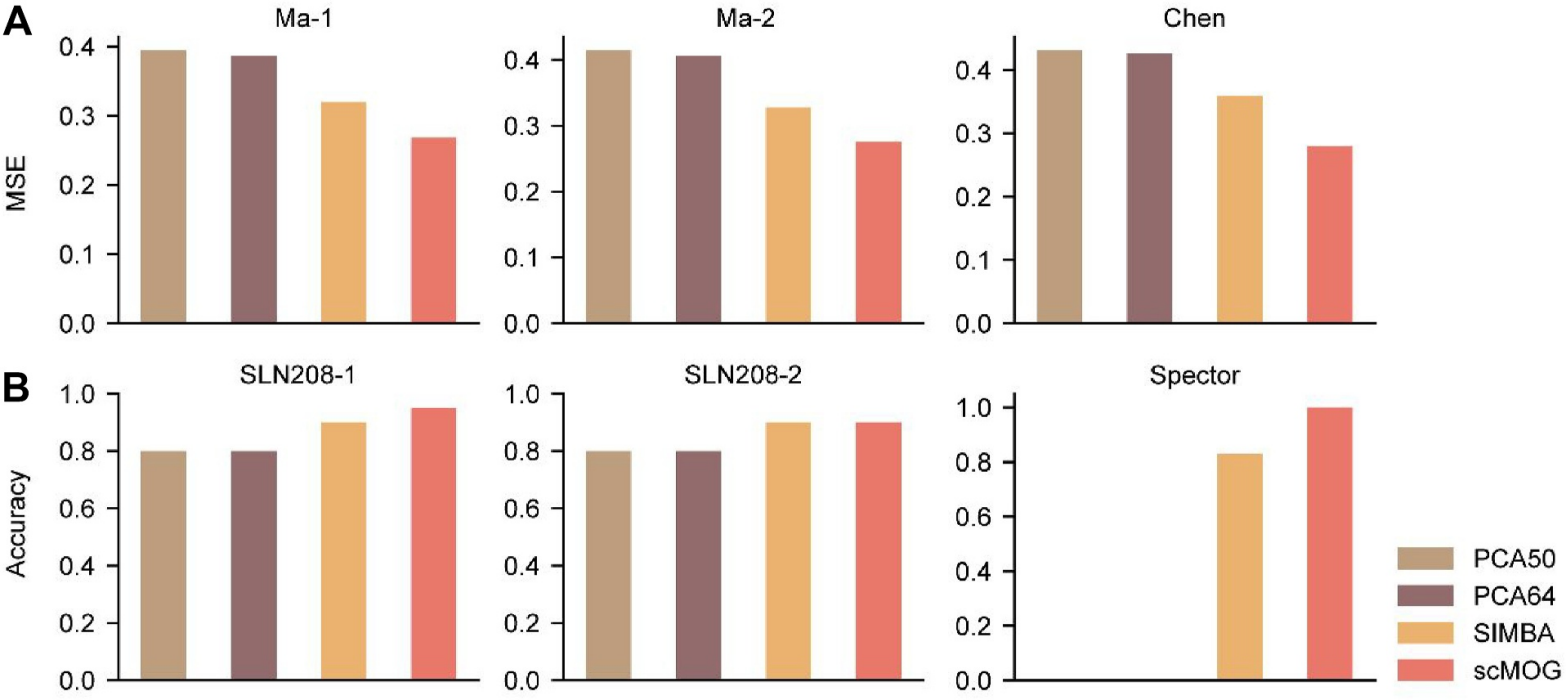
Comprehensive comparison of different methods for preserving transcriptional regulatory and protein–protein interaction information. (A) Mean squared error (MSE) values calculated using different methods on the Ma‐1, Ma‐2, and Chen datasets. (B) Accuracy values calculated using different methods on the SLN208‐1, SLN208‐2, and Spector datasets. Missing values indicate that the corresponding method could not compute the respective metric.

By comparing MSE values, this experiment further verifies scMOG’s superiority in preserving gene–peak regulatory relationships and highlights its effectiveness in integrative analysis.

### 2.5 Preserving similarity between protein pairs

Intracellular signaling responses and protein regulatory mechanisms are critical for biological research and clinical applications. Accurately predicting protein–protein interactions (PPIs) helps reveal fundamental biological functions [45]. Protein interactions often rely on structural similarity or functional relevance between proteins [46]. Therefore, computing protein similarity can serve as an indirect approach to infer potential interactions. Because scMOG can obtain protein embeddings when processing multi‐omics datasets that include proteomics, we leverage these embeddings to identify interacting protein pairs.

Specifically, we first preprocess protein names to ensure consistency between experimental data and the real protein names recorded in the STRING database. To ensure a fair comparison, we take the intersection of proteins retained after preprocessing across all methods. Then, based on the filtered protein embeddings, we construct all possible protein pairs and compute the similarity between each pair. By sorting these similarity values in descending order, we focus on the top 2% of high‐similarity protein pairs. These highly similar protein pairs are then compared with known interactions in the STRING database to evaluate the predictive performance of different feature embeddings.

We performed experiments on three multi‐omics datasets containing proteomics and compared scMOG with SIMBA, PCA50, and PCA64. By identifying the top 2% of high‐similarity protein pairs and comparing them against known interacting pairs in the STRING database, we evaluated the predictive power of the learned embeddings. On the Spector dataset, scMOG successfully identified interacting protein pairs that SIMBA failed to detect, such as CD28, SELL. Further investigation in the STRING database revealed that both CD28 and SELL are associated with the KEGG pathway HSA04514 (cell adhesion molecules). Similarly, on the SLN208‐1 dataset, scMOG identified interacting protein pairs such as ITGAL and TNFRSF9, which SIMBA failed to detect. Further analysis showed that this protein pair is significantly associated with biological phenotypes in the Mammalian Phenotype Ontology (Monarch). On the SLN208‐2 dataset, scMOG identified (Ccr4, Ms4a1) as an interacting protein pair, which SIMBA did not detect. According to the STRING database, this pair is enriched in the MMU‐202733 pathway, which is related to cell surface interactions at the vascular wall. Regarding performance metrics, Figure 4B shows that scMOG achieved a higher accuracy score than SIMBA on the Spector and SLN208‐1 datasets, whereas its accuracy score on the SLN208‐2 dataset was comparable to SIMBA’s, further confirming the biological relevance of the protein embeddings learned by scMOG.

Through this experiment, we verified that scMOG effectively captures protein–protein interactions, demonstrating its advantage in multi‐omics data integration.

### 2.6 Differential expression analysis

We identified differentially expressed genes (DEGs) for each cell cluster using the nonparametric Wilcoxon rank‐sum test. These genes can serve as marker genes for specific cell types. We selected the top three DEGs from each cluster and visualized their expression patterns. The bubble plot in Figure S9A illustrates the expression levels of DEGs identified from the PBMC‐2 dataset across different clusters. The size of each bubble represents the proportion of cells in a given cluster expressing the gene, whereas the color indicates the average expression level within the cluster. We analyzed the identified DEGs using the CellMarker database [47] and found that the cell clusters predicted by scMOG preserved true differential expression patterns, aiding in cell type annotation. For example, *GNLY* and *NKG7*, identified as DEGs in Cluster 8, are well‐known marker genes for natural killer cells. In Cluster 2, *LEF1*, *FHIT*, and *CAMK4* are marker genes for naive CD8^+^ T cells, whereas *TCF7L2* and *PSAP*, identified in Cluster 7, are marker genes for CD1C‐CD141‐dendritic cells. Although some genes did not match the known DEGs in the CellMarker database, visualization revealed that certain genes were highly expressed in specific clusters. As shown in Figure S9B, *A2M*, *BANK1*, and *RALGPS2* exhibited strong expression in particular cell clusters, suggesting their potential role as novel marker genes. The identification of these DEGs provides important insights into the heterogeneity of cell populations and their potential gene regulatory mechanisms.

### 2.7 Ablation study and hyperparameter analysis

To evaluate the contributions of different modules in scMOG, we conducted an ablation study, which included replacing the encoder with an MLP, removing the contrastive learning module, and removing the omics alignment module. In the experiment where the encoder was replaced with an MLP, we kept all other parameters and network structures unchanged, ensuring that the encoders remained a two‐layer network. When ablating the contrastive learning module and omics alignment module, we removed the corresponding loss terms from the loss function while keeping the model architecture unchanged. We conducted experiments on eight multi‐omics datasets used in this study and visualized the evaluation metrics as boxplots (Figure 5). The results demonstrate that the complete scMOG model achieves the best performance in ARI, NMI, and ACC, verifying the importance of the encoder, contrastive learning module, and omics alignment module in data integration. As shown in Figure 5A, when the encoder was replaced with an MLP, the performance of scMOG dropped significantly, confirming that the designed LightGCN‐based encoder contributes to the model’s superiority.

**FIGURE 5:**
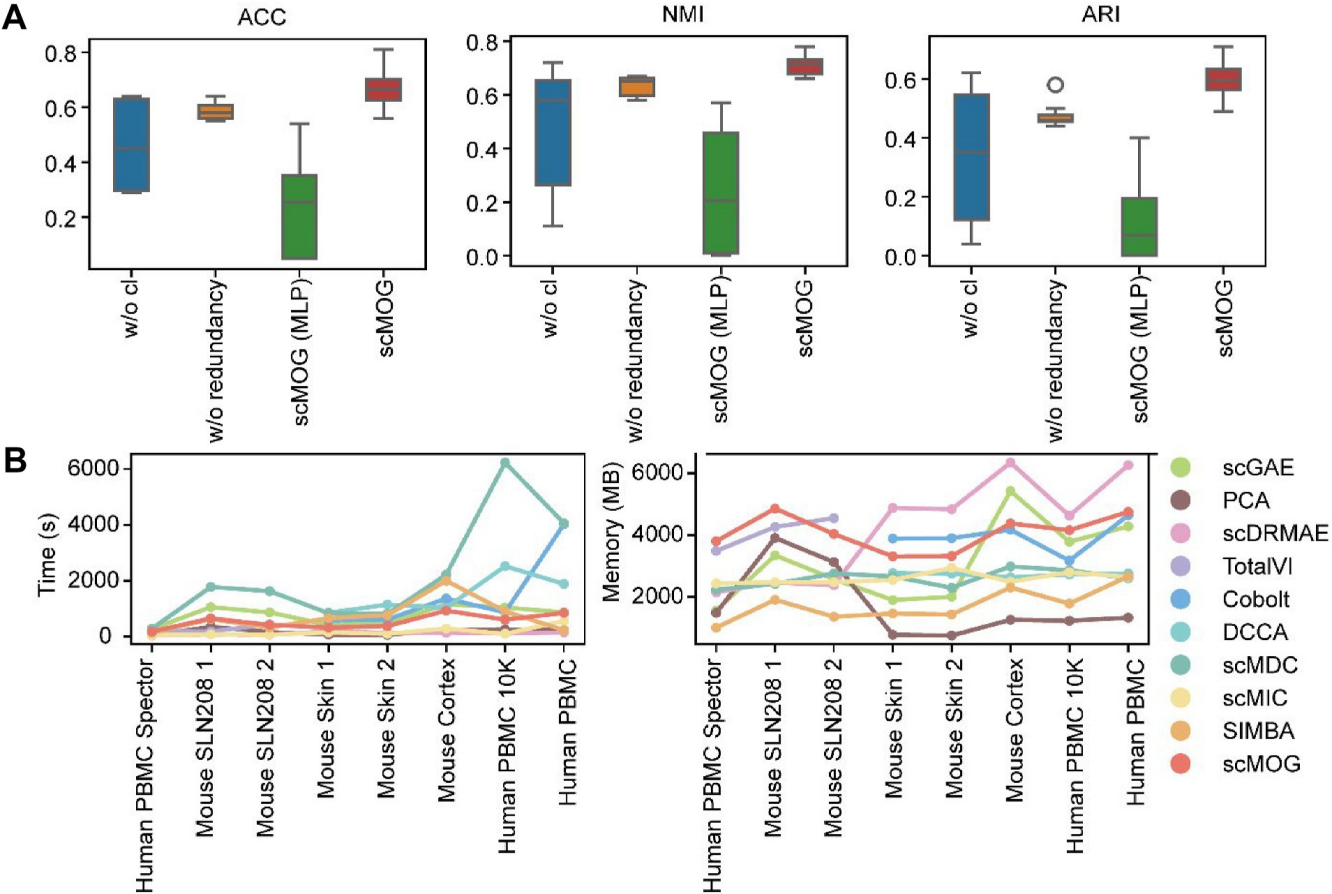
Box plots of ablation experiments and line plots of computation time and memory usage. (A) Box plots illustrating the results of ablation experiments. Each box plot represents a specific evaluation metric, where the blue box corresponds to the removal of the contrastive learning module, the orange box to the removal of the omics alignment module, the green box to replacing the encoder with an MLP, and the red box to the complete version of scMOG with all modules included. Each box contains metric values computed from the eight datasets used in this study. (B) Line plots of computation time and memory usage. The left plot shows the computation time of scMOG and comparison algorithms across the eight datasets, whereas the right plot illustrates their memory consumption. ACC, accuracy; ARI, adjusted Rand index; NMI, normalized mutual information.

To assess the performance of scMOG under different hyperparameter settings, we conducted experiments on the eight datasets. First, for each dataset, we fixed the omics alignment module loss ratio and clustering resolution, kept all other hyperparameters unchanged, and tested different contrastive learning loss ratios. The results, shown in Figure S10A, indicate that the default setting of scMOG (cl_ratio = 1) achieves the highest ACC, NMI, and ARI. Next, for each dataset, we selected the optimal contrastive learning loss ratio from the previous step, kept all other hyperparameters unchanged, and tested different omics alignment module loss ratios. As shown in Figure S10B, the default scMOG setting (alignment_ratio = 1) consistently achieved the highest average ACC, NMI, and ARI across all eight datasets. Finally, using the optimal contrastive learning loss ratio and omics alignment module loss ratio obtained in the previous steps while keeping other hyperparameters fixed, we tested various Louvain clustering resolutions. The results shown in Figure S10C indicate that under the default setting (resolution = 0.6), scMOG consistently achieved the highest average ACC, NMI, and ARI across all eight datasets.

In conclusion, the ablation study results highlight that the combination of the LightGCN‐based encoder, omics alignment module, and contrastive learning module is crucial for the superior performance of scMOG in multi‐omics data integration. The hyperparameter analysis further demonstrates that scMOG performs optimally under its default hyperparameter settings, validating the model’s robustness and stability.

### 2.8 Computation time and memory usage

We compared the runtime and memory consumption of scMOG with nine other methods on eight real datasets. These datasets include cell type annotations from mouse and human, specifically, Ma‐1, Ma‐2, PBMC‐1, PBMC‐2, Chen, Spector, SLN208‐1, and SLN208‐2 (Table 1). The datasets vary in size and complexity, with the number of cells ranging from 3000 to over 10,000.

Across datasets of varying scales, scMOG demonstrates efficiency by generally ranking in the mid‐to‐lower range in terms of computation time compared to other methods. Notably, when processing the Mouse Skin 1 and Mouse Skin 2 datasets, scMOG required significantly less computation time than several other methods, such as scGAE and DCCA, indicating its computational efficiency on these datasets. For larger datasets such as human PBMC 10K and human PBMC, the computation time of scMOG increased slightly but remained at a moderate level, without experiencing a sharp rise. This further confirms that scMOG can efficiently handle large‐scale datasets while avoiding excessive computational overhead.

Regarding memory usage, scMOG has a relatively high peak memory consumption, but it remains within the same order of magnitude as other deep learning‐based methods. On larger datasets such as human PBMC 10K and human PBMC, the memory consumption of scMOG increases, which aligns with the characteristics of large‐scale data processing. Although scMOG ranks as the second highest in memory usage, it still consumes less memory than scDRMAE, another deep learning‐based method. Despite its higher memory usage, scMOG remains within an acceptable range, and for most datasets, its memory consumption is reasonable compared to other methods. For cases with limited computing resources, we recommend training scMOG on smaller subgraphs, which can effectively reduce memory overhead and accelerate training, optimizing memory usage while maintaining high performance.

Overall, scMOG achieves a balance between computation time and memory efficiency, demonstrating high efficiency and stability when processing large‐scale multi‐omics datasets. Although its memory consumption is relatively high, its computational efficiency and scalability make it a viable option, especially for studies that require high integration performance.

## 3 DISCUSSION AND CONCLUSION

This study proposes scMOG, a method based on a heterogeneous GNN architecture that integrates cross‐modal information between cell nodes and molecular feature nodes to accurately depict the cellular heterogeneity landscape of paired omics samples (transcriptomics and epigenomics, transcriptomics and proteomics). scMOG models the complex relationships between omics layers while preserving biologically meaningful low‐dimensional representations within an autoencoder framework. It incorporates a contrastive learning module and an omics alignment module, effectively capturing shared patterns across omics layers while maintaining layer‐specific features. Unlike conventional methods that simply generate integrated cell representations, scMOG enables bidirectional guidance between omics, allowing it to perceive correlations between features. Experimental results demonstrate that scMOG exhibits significant advantages not only in cell clustering and visualization but also in capturing intrinsic cross‐omics regulatory and feature correlations, providing a robust framework for identifying potential marker genes and supporting deep biological insight.

First, from an overall architectural perspective, scMOG constructs a heterogeneous graph between cells and features, explicitly modeling the potential interactions across different omics layers. Traditional methods often rely on techniques such as matrix factorization or manifold alignment, which struggle to balance the heterogeneity and complementarity of multi‐omics data. In contrast, scMOG employs a GNN encoder (Figure 1B) to capture the topological relationships between cells and molecular features. By leveraging multilayer convolutions and weighted summation, scMOG effectively prevents oversmoothing, leading to more discriminative low‐dimensional embeddings. Second, the model addresses data sparsity by incorporating the Hadamard product with the ZINB decoders. The ZINB loss function constrains the reconstructed results to maintain a high degree of consistency with the true data distribution. Additionally, scMOG incorporates a contrastive learning module with a triplet loss function to pull similar cells closer together in the low‐dimensional space while pushing dissimilar cells apart. Experimental results (Figure 5A) show that this contrastive learning mechanism significantly improves the model’s performance. Furthermore, scMOG employs an omics alignment module that adaptively strengthens shared cross‐omics structure, ensuring that the features in the shared space faithfully represent the intrinsic commonalities of cells.

Experimental results on eight real datasets further validate the superiority of scMOG. When integrating transcriptomics and epigenomics, scMOG effectively separates different cell types with clear boundaries. In addition, clustering performance metrics indicate that scMOG outperforms existing methods such as PCA, scGAE, and DCCA on most datasets. In the task of integrating transcriptomics and proteomics, scMOG also demonstrates outstanding performance. Further analysis of protein embeddings reveals that scMOG successfully identifies key interacting protein pairs known in the STRING database, providing crucial support for subsequent cellular signaling pathway analysis and molecular mechanism studies. Furthermore, to comprehensively evaluate the contribution of each module to the overall performance of scMOG, we conducted detailed ablation studies (Figure 5A). In these experiments, we replaced the encoder with a simple MLP, removed the contrastive learning module, and removed the omics alignment module, each leading to a significant decline in performance. These results indicate that the LightGCN‐based encoders play a key role in capturing the topological relationships between cells and features. Additionally, both the contrastive learning module and the omics alignment module are essential for enhancing model robustness and optimizing low‐dimensional embeddings. With the synergistic interaction of these modules, scMOG achieves efficient information fusion, providing a more reliable foundation for biological function discovery.

Although scMOG demonstrates strong integration capabilities in experiments, there are still several limitations that require further improvement. First, scMOG is specifically designed for cell‐by‐cell paired multi‐omics data because the omics alignment module relies on cell‐level correspondence to calculate cross‐omics similarity. However, truly paired single‐cell multi‐omics datasets remain relatively scarce, costly, and technically challenging to obtain. This limits the direct applicability of scMOG to a broader range of single‐cell studies. In future work, we plan to explore possible extensions of scMOG to unmatched datasets, for example, by incorporating MNN matching strategies to approximate cross‐omics cell correspondence. Such adaptation may expand scMOG’s applicability to more realistic experimental settings. Second, when handling large‐scale single‐cell datasets, scMOG exhibits high memory consumption (Figure 5B), which may pose challenges in resource‐limited computational environments. To address this, future work could explore subgraph sampling, sparse matrix computation, and other techniques to reduce memory usage and improve computational efficiency. Third, although scMOG works well for transcriptomic, epigenomic, and proteomic integration, its generalizability to other omics layers remains to be tested. In future work, we will extend scMOG and explore the use of decoders tailored to each modality to maintain effective information fusion in more complex scenarios.

## 4 MATERIALS AND METHODS

In this section, we will provide a detailed description of the fundamental architecture of scMOG, including data preprocessing, the graph autoencoder, the contrastive learning module, and the omics alignment module. Additionally, we will explain the experimental setup.

### 4.1 Data preprocessing

We use the Scanpy package [40] to preprocess multi‐omics data. For gene expression data, we first filter out low‐quality genes expressed in fewer than three cells. Next, we normalize the data based on the library size of each cell, followed by a logarithmic transformation. Finally, we select the top 3000 highly variable genes as input. For chromatin accessibility data, scMOG adopts the latent semantic indexing (LSI) algorithm [48, 49] for preprocessing. Similarly, we select the top 3000 highly variable chromatin regions as input. To construct positive sample pairs required for contrastive learning, we apply PCA for dimensionality reduction on gene expression data and use the “LSI” function for dimensionality reduction on chromatin accessibility data. The preprocessing steps for the benchmark methods are detailed in Table S3.

### 4.2 Graph construction

To represent the relationships between cells and features in different omics, scMOG uses a GNN as the encoder. Based on the preprocessed data matrices $X_{1}^{\prime }$ and $X_{2}^{\prime }$, a bipartite graph is constructed as follows: $G_{i}=\left(C_{i},F_{i},A_{i}\right)$, i=1,2, where $C_{i}$ represents the set of cells in the i‐th omics, $F_{i}$ represents the set of features (genes, chromatin regions, or proteins) in the i‐th omics, and $A_{i}$ represents the adjacency matrix of the i‐th omics. For the i‐th omics, its adjacency matrix is constructed as follows:

(1) $$A_{i}=\left(\begin{array}{cc} O_{1}^{i} & R_{i}\\ R_{i}^{T} & O_{2}^{i} \end{array}\right),$$

where $O_{1}^{i}{\in R}^{n\times n}$ and $O_{2}^{i}{\in R}^{m_{i}\times m_{i}}$ are zero matrices. The matrix $R_{i}=\left[r_{jk}^{i}\right]{\in R}^{n\times m_{i}}$ captures the relationships between cells and features, defined as follows:

(2) $$r_{jk}^{i}=\left\{\begin{array}{cc} 1, & \text{if}\;\text{the}\;\text{value}\;\text{of}\;\text{feature}\;k\;\text{in}\;\text{cell}\;j\;\text{is}\;\text{non}-\text{zero},\\ 0, & \text{otherwise}.\; \end{array}\right.$$

In summary, for each omics layer, we construct a distinct heterogeneous graph, denoted as $G=\left\{G_{i}|i=1,2\right\}$, where nodes represent cells and features, and edges represent relationships between them.

For the case of integrating gene expression and chromatin accessibility data, scMOG follows the single‐cell multi‐omics integration method SIMBA [31], constructing the adjacency matrix $A_{f}=\left[a_{ij}^{f}\right]{\in R}^{m_{1}\times m_{2}}$ between genes and peaks. Based on gene annotations for humans (hg38) and mice (mm10), if a peak region overlaps with a gene body or is located within 5 kb upstream of a gene body, the peak region is considered a potential regulatory element of gene expression. The adjacency matrix is then defined as follows:

(3) $$a_{ij}^{f}=\left\{\begin{array}{cc} 1, & \text{if}\;\text{gene}\;i\;\text{is}\;\text{neighboring}\;\text{to}\;\text{peak}\;j,\\ 0, & \text{otherwise}. \end{array}\right.$$

### 4.3 Graph encoder

Assume that we have obtained heterogeneous graphs for two omics layers and use a GNN to extract cell and feature representations from the graphs. First, we initialize the embeddings for cell j and feature k in the i‐th omics as $e_{c_{i},j}^{\left(0\right)}{\in R}^{d}$ and $e_{f_{i},k}^{\left(0\right)}{\in R}^{d}$, respectively. To improve clarity in the workflow, we use $e_{g,k}^{\left(0\right)}$ for the initial embedding of genes and $e_{p,k}^{\left(0\right)}$ for the initial embedding of peaks. The initial embeddings are updated through network training. The convolution at layer l is computed as follows:

(4) $$e_{c_{i},j}^{\left(l\right)}=\text{Aggregate}\left\{e_{f_{i},k}^{\left(l-1\right)}|k\in N_{c_{i},j}\right\}=\sum_{k\in N_{c_{i},j}}\frac{1}{\sqrt{\left|N_{c_{i},j}\right|\left|N_{f_{i},k}\right|}}e_{f_{i},k}^{\left(l-1\right)},i=1,2.$$

Similarly, feature embeddings are updated as follows:

(5) $$e_{f_{1},k}^{\left(l\right)}=\text{Aggregate}\left\{e_{c_{1},i}^{\left(l-1\right)},e_{f_{2},j}^{\left(l-1\right)}|i\in N_{f_{1},k},j\in N_{f_{1},k}^{\prime }\right\}=\sum_{i\in N_{f_{1},k}}\frac{1}{\sqrt{\left|N_{f_{1},k}\|N_{c_{1},i}\right|}}e_{c_{1},i}^{\left(l-1\right)}+\sum_{j\in N_{f_{1},k}^{\prime }}e_{f_{2},j}^{\left(l-1\right)},$$

(6) $$e_{f_{2},k}^{\left(l\right)}=\text{Aggregate}\left\{e_{c_{2},i}^{\left(l-1\right)},e_{f_{1},j}^{\left(l-1\right)}|i\in N_{f_{2},k},j\in N_{f_{2},k}^{\prime }\right\}=\sum_{i\in N_{f_{2},k}}\frac{1}{\sqrt{\left|N_{f_{2},k}\|N_{c_{2},i}\right|}}e_{c_{2},i}^{\left(l-1\right)}+\sum_{j\in N_{f_{2},k}^{\prime }}e_{f_{1},j}^{\left(l-1\right)},$$

where l=1,2,⋯,L, and L denotes the total number of convolutional layers. $N_{c_{i},j}$ represents the set of neighboring features for cell j in the i‐th omics, $N_{f_{i},k}$ represents the set of neighboring cells for feature k in the i‐th omics, and $N_{f_{i},k}^{\prime }$ represents the set of neighboring features for feature k in the i‐th omics.

By applying L layers of convolution, we obtain L+1 sets of low‐dimensional embeddings for both cells and features in each omics. To mitigate oversmoothing caused by convolution and enhance the encoder’s ability to extract information, we apply a weighted summation to these embeddings to obtain the final embeddings. The computation is formulated as follows:

(7) $$e_{c_{i},j}=\sum_{l=0}^{L}w_{c_{i}}^{\left(l\right)}e_{c_{i},j}^{\left(l\right)},$$

(8) $$e_{f_{i},k}=\sum_{l=0}^{L}w_{f_{i}}^{\left(l\right)}e_{f_{i},k}^{\left(l\right)},$$

where $e_{c_{i},j}$ denotes the final embedding of cell j, and $e_{f_{i},k}$ denotes the final embedding of feature k. $w_{c_{i}}^{\left(l\right)}$ and $w_{f_{i}}^{\left(l\right)}$ are learnable parameters of the network. Importantly, the embeddings of cells and features are not learned independently. Both $e_{c_{i},j}$ and $e_{f_{i},k}$ are jointly optimized within the same GNN encoder and under the same training objectives.

### 4.4 Decoder

To enhance the encoding capability of the encoder and retain more biological information in the low‐dimensional embeddings, scMOG adopts the ZINB decoder to reconstruct the heterogeneous graph G. The ZINB distribution is formulated as follows:

(9) ZINB(x;μ,θ,π)=πδ(x)+(1−π)NB(x;μ,θ),

where μ,θ,π are three parameters of the ZINB distribution, representing the mean, dispersion, and zero‐inflation factor, respectively. δ(x) denotes the Dirac delta function, and NB(x;μ,θ) represents the probability mass function of the NB distribution, given by the following equation:

(10) $$\text{NB}\left(x;\mu ,\theta \right)=\frac{\Gamma \left(x+\theta \right)}{\Gamma \left(\theta \right)\Gamma \left(x+1\right)}{\left(\frac{\theta }{\theta +\mu }\right)}^{\theta }{\left(\frac{\mu }{\theta +\mu }\right)}^{x}.$$

The ZINB decoder used in scMOG incorporates the idea of NMF. For each omics layer, the Hadamard product is first applied to cell embeddings and feature embeddings, as shown in Equation (11). Then, three fully connected networks are used to estimate the three parameters μ,θ,π of the ZINB distribution, as formulated in Equation (11):

(11) $$\left\{\begin{array}{c} \begin{array}{c} e_{jk}^{i}=e_{c_{i},j}⊙e_{f_{i},k},\\ \mu_{jk}^{i}=\exp\left(\text{sigmoid}\left(W_{\mu }^{i}e_{jk}^{i}+b_{\mu }^{i}\right)\times s_{k}^{i}\right)\times l_{j}^{i}, \end{array}\\ \begin{array}{c} \theta_{jk}^{i}=\text{softplus}\left(\left(W_{\theta }^{i}e_{jk}^{i}+b_{\theta }^{i}\right)\times s_{k}^{i}\right),\\ \pi_{jk}^{i}=\text{sigmoid}\left(W_{\pi }^{i}e_{jk}^{i}+b_{\pi }^{i}\right), \end{array} \end{array}\right.$$

where $s_{k}^{i}$ and $l_{j}^{i}$ represent scaling factors. The learnable weight matrices are given by $W_{\mu }^{i}{\in R}^{1\times d}$, $W_{\theta }^{i}{\in R}^{1\times d}$, and $W_{\pi }^{i}{\in R}^{1\times d}$. Additionally, $b_{\mu }^{i}\in R$, $b_{\theta }^{i}\in R$, and $b_{\pi }^{i}\in R$ are learnable biases.

### 4.5 Contrastive learning module

To facilitate the integration of multi‐omics data while preserving the unique characteristics of each omics, scMOG employs contrastive learning to extract low‐dimensional embeddings of both cells and features. Specifically, after preprocessing, the data matrices $X_{1}^{\prime }$ and $X_{2}^{\prime }$ are reduced using PCA and “LSI” functions, respectively. In the processed space, the KD‐tree algorithm [50] is utilized to find *k*‐nearest neighbors for each omics. If, in the i‐th omics, cell a and cell p are *k*‐nearest neighbors, they form an MNN pair $\left(a_{i},p_{i}\right)$. All MNN pairs within each omics constitute the set of positive samples. For each MNN pair, a random cell is selected as a negative sample, forming a triplet set $S_{i}=\left\{\left(a_{i},p_{i},n_{i}\right)\right\}$. The contrastive learning module employs a triplet loss function, formulated as follows:

(12) $$L_{CL}^{i}=\sum_{\left(a_{i},p_{i},n_{i}\right)\in S_{i}}\max\left({∥e_{c_{i},a}-e_{c_{i},p}∥}_{2}-{∥e_{c_{i},a}-e_{c_{i},n}∥}_{2}+m,0\right),$$

where m is a hyperparameter. If the distance between the anchor and the positive sample, plus a margin m, exceeds the distance between the anchor and the negative sample, the loss becomes non‐zero. By optimizing this loss function, the model learns to pull similar cells closer in the embedding space while pushing apart dissimilar ones.

### 4.6 Omics alignment module

The autoencoder structures for each omics described above operate in parallel and require module connections. Although different omics provide complementary biological information, representations derived from separate omics layers for the same cells should remain consistent in the shared embedding space. To enhance cross‐omics coherence and ensure that matched cells are aligned in the latent space, scMOG introduces an omics alignment loss, formulated as follows:

(13) $$L_{\text{alignment}}={\left\|\frac{E_{c_{1}}\cdot E_{c_{2}}^{T}}{∥E_{c_{1}}{∥}_{2}\cdot ∥E_{c_{2}}{∥}_{2}}-I\right\|}_{2}^{2},$$

where $I{\in R}^{n\times n}$ represents an identity matrix with diagonal elements of 1 and all other elements being 0. $E_{c_{1}}$ and $E_{c_{2}}$ denote the cell representations of transcriptomic data and epigenomic data, respectively.

### 4.7 Loss function

To reduce memory consumption during training, the heterogeneous graph $G_{i}\left(i=1,2\right)$ of each omics is sampled by preserving 10% of the edges that exist and do not exist between cell and feature nodes. These sampled edges serve as inputs to the ZINB decoder. scMOG employs a collaborative graph autoencoder for each omics, training the network by minimizing the negative log‐likelihood function. The reconstruction loss for the i‐th omics is formulated as follows:

(14) $$L_{\text{ZINB}}^{i}=-\frac{1}{N}\sum_{j,k}\ln\text{ZINB}\left(x_{jk}^{i}|\mu_{jk}^{i},\theta_{jk}^{i},\pi_{jk}^{i}\right),$$

and the overall reconstruction loss is given by the following equation:

(15) $$L=\sum_{i=1}^{2}L_{\text{ZINB}}^{i},$$

where $x_{jk}^{i}$ represents the observed count value of the k‐th feature in the j‐th cell of the i‐th omics, and N denotes the total number of sampled edges.

To reduce overfitting, scMOG introduces a regularization term $L_{\text{reg}}$ to constrain the initialization of cell embeddings and feature embeddings. The regularization term for the i‐th omics is defined in Equation (16), and the total regularization loss is given in Equation (17):

(16) $$L_{\text{reg}}^{i}=\frac{{∥E_{c_{i}}^{\left(0\right)}∥}_{F}^{2}+{∥E_{f_{i}}^{\left(0\right)}∥}_{F}^{2}}{2\left(n+m_{i}\right)},$$

(17) $$L_{\text{reg}}=\sum_{i=1}^{2}L_{\text{reg}}^{i},$$

where $E_{c_{i}}^{\left(0\right)}$ and $E_{f_{i}}^{\left(0\right)}$ denote the initial embeddings of cells and features in the i‐th omics, respectively, and ${\left\|\cdot \right\|}_{F}$ represents the Frobenius norm.

The total loss of the contrastive learning module is defined in Equation (18). Thus, the overall loss function of scMOG is formulated as Equation (19):

(18) $$L_{\text{CL}}=\sum_{i=1}^{2}L_{\text{CL}}^{i},$$

(19) $$L=L_{\text{ZINB}}+\alpha L_{\text{CL}}+\beta L_{\text{alignment}}+\gamma L_{\text{reg}},$$

where α, β, and γ are hyperparameters used to balance different loss terms.

### 4.8 Implementation details

We implement scMOG using PyTorch 1.13.1 in the Python 3.9.17 environment, and all experiments are conducted on the Linux system. scMOG can simultaneously take as input transcriptomics and epigenomics or transcriptomics and proteomics. During data preprocessing, the number of highly variable features is set to 3000. The encoding layers of the autoencoder are configured as (3000, 64, 64), and the shared space dimension is set to 64. The ZINB decoder first applies Hadamard product operations, reducing the feature dimension from 64 to 1. The transcriptomic data finds *k*‐nearest neighbors in PCA space, with the PCA dimension set to 50 and the number of *k*‐nearest neighbors set to 20. Contrastive learning selects 10% of MNN pairs in each iteration to calculate the contrastive loss, with the hyperparameter m for contrastive loss set to 2. The total loss is balanced by the hyperparameters α, β, and γ, where both α and β are set to 1, and γ is set to 0.0001. The Adam optimizer [51] is used for training, with a learning rate of 0.1. We use the Louvain [52] algorithm for clustering. When the input includes transcriptomics and epigenomics, the resolution is set to 0.6; otherwise, the resolution is set to 1.0. All contrastive calculations follow the implementations in the corresponding cited papers, with hyperparameters set according to their default configurations. Because scMOG consistently performs well across different datasets under default settings, we recommend that users execute scMOG with the default parameters.

### 4.9 Data sources

This study utilizes eight real datasets with cell type annotations, derived from mice or humans, which can be downloaded via GSM numbers or from 10x Genomics. Specifically, datasets Ma‐1 and Ma‐2 were provided by Ma et al. [53]. The datasets PBMC‐1 [31], PBMC‐2 [54], and Spector [41] were all sourced from 10x Genomics. The dataset Chen originates from the mouse epidermis data provided by Chen et al. [55]. Additionally, datasets SLN208‐1 and SLN208‐2 were provided by Gayoso et al. [24].

## AUTHOR CONTRIBUTIONS

**Yucheng Lu**: Methodology; data curation; formal analysis; writing—original draft; writing—review and editing. **Xun Zhang**: Data curation; formal analysis. **Hongwei Li**: Supervision; conceptualization; funding acquisition; resources.

## CONFLICT OF INTEREST STATEMENT

The authors declare no conflicts of interest.

## ETHICS STATEMENT

This article does not contain any studies with human or animal materials performed by any of the authors.

## Supporting information

Supporting Information S1

## Data Availability

The source codes are available at the GitHub website (luyucheng01/scMOG_master).

## References

[1] Barakat R , Chatterjee J , Mu R , Qi X , Gu X , Smirnov I , et al. Human single cell RNA‐sequencing reveals a targetable CD8^+^ exhausted T cell population that maintains mouse low‐grade glioma growth. Nat Commun. 2024;15(1):1–16. 10.1038/s41467-024-54569-4 PMC11605098 39609412

[2] Yin K , Xu Y , Guo Y , Zheng Z , Lin X , Zhao M , et al. Dyna‐vivo‐seq unveils cellular RNA dynamics during acute kidney injury via in vivo metabolic RNA labeling‐based scRNA‐seq. Nat Commun. 2024;15(1):9866. 10.1038/s41467-024-54202-4 PMC11564529 39543112

[3] Tegowski M , Prater AK , Holley CL , Meyer KD . Single‐cell m^6^A profiling in the mouse brain uncovers cell type‐specific RNA methylomes and age‐dependent differential methylation. Nat Neurosci. 2024;27(12):2512–2520. 10.1038/s41593-024-01768-3 PMC11614689 39317796

[4] Wu X , Yang X , Dai Y , Zhao Z , Zhu J , Guo H , et al. Single‐cell sequencing to multi‐omics: technologies and applications. Biomark Res. 2024;12(1):110. 10.1186/s40364-024-00643-4 PMC11438019 39334490

[5] Liu L , Liu C , Quintero A , Wu L , Yuan Y , Wang M , et al. Deconvolution of single‐cell multi‐omics layers reveals regulatory heterogeneity. Nat Commun. 2019;10(1):470. 10.1038/s41467-018-08205-7 PMC6349937 30692544

[6] Baysoy A , Bai Z , Satija R , Fan R . The technological landscape and applications of single‐cell multi‐omics. Nat Rev Mol Cell Biol. 2023;24(10):695–713. 10.1038/s41580-023-00615-w PMC10242609 37280296

[7] Eraslan G , Simon LM , Mircea M , Mueller NS , Theis FJ . Single‐cell RNA‐seq denoising using a deep count autoencoder. Nat Commun. 2019;10(1):390. 10.1038/s41467-018-07931-2 PMC6344535 30674886

[8] Qian K , Fu S , Li H , Li WV . scINSIGHT for interpreting single‐cell gene expression from biologically heterogeneous data. Genome Biol. 2022;23(1):82. 10.1186/s13059-022-02649-3 PMC8935111 35313930

[9] He X , Qian K , Wang Z , Zeng S , Li H , Li WV . scAce: an adaptive embedding and clustering method for single‐cell gene expression data. Bioinformatics. 2023;39(9):btad546. 10.1093/bioinformatics/btad546 PMC10500084 37672035

[10] Wang X , Yang S , Li H . scSCC: a swapped contrastive learning‐based clustering method for single‐cell gene expression data. Quant Biol. 2025;13(2):e85. 10.1002/qub2.85 PMC12806040 41675506

[11] Ren L , Wang J , Li Z , Li Q , Yu G . scMCs: a framework for single‐cell multi‐omics data integration and multiple clusterings. Bioinformatics. 2023;39(4):btad133. 10.1093/bioinformatics/btad133 PMC10070040 36929930

[12] Shi Z , Cong L , Wu H . MomicPred: a cell cycle prediction framework based on dual‐branch multi‐modal feature fusion for single‐cell multi‐omics data. IEEE J Biomed Health Inform. 2026;30(3):2242–51. 10.1109/JBHI.2025.3595904 40768465

[13] Yang X , Mann KK , Wu H , Ding J . scCross: a deep generative model for unifying single‐cell multi‐omics with seamless integration, cross‐modal generation, and in silico exploration. Genome Biol. 2024;25(1):198. 10.1186/s13059-024-03338-z PMC11285326 39075536

[14] Altay A , Vingron M . scATAcat: cell‐type annotation for scATAC‐seq data. NAR Genom. Bioinform. 2024;6(4):lqae135. 10.1093/nargab/lqae135 PMC11459382 39380946

[15] Lynch AW , Theodoris CV , Long HW , Brown M , Liu XS , Meyer CA . MIRA: joint regulatory modeling of multimodal expression and chromatin accessibility in single cells. Nat Methods. 2022;19(9):1097–1108. 10.1038/s41592-022-01595-z PMC9517733 36068320

[16] Argelaguet R , Velten B , Arnol D , Dietrich S , Zenz T , Marioni JC , et al. Multi‐omics factor analysis—a framework for unsupervised integration of multi‐omics data sets. Mol Syst Biol. 2018;14(6):e8124. 10.15252/msb.20178124 PMC6010767 29925568

[17] Duren Z , Chen X , Zamanighomi M , Zeng W , Satpathy AT , Chang HY , et al. Integrative analysis of single‐cell genomics data by coupled nonnegative matrix factorizations. Proc Natl Acad Sci. 2018;115(30):7723–7728. 10.1073/pnas.1805681115 PMC6065048 29987051

[18] Welch JD , Hartemink AJ , Prins JF . MATCHER: manifold alignment reveals correspondence between single cell transcriptome and epigenome dynamics. Genome Biol. 2017;18(1):1–19. 10.1186/s13059-017-1269-0 PMC5525279 28738873

[19] Zhou X , Wu H . scHiClassifier: a deep learning framework for cell type prediction by fusing multiple feature sets from single‐cell Hi‐C data. Briefings Bioinf. 2024;26(1):bbaf009. 10.1093/bib/bbaf009 PMC11744636 39831891

[20] Wu Y , Shi Z , Zhou X , Zhang P , Yang X , Ding J , et al. scHiCyclePred: a deep learning framework for predicting cell cycle phases from single‐cell Hi‐C data using multi‐scale interaction information. Commun Biol. 2024;7(1):923. 10.1038/s42003-024-06626-3 PMC11291681 39085477

[21] Li L , Li X , Wu H . A novel deep learning framework with dynamic tokenization for identifying chromatin interactions along with motif importance investigation. Briefings Bioinf. 2025;26(3):bbaf289. 10.1093/bib/bbaf289 PMC12204613 40536817

[22] Kingma DP , Welling M . Auto‐encoding variational bayes. arXiv. 2013.

[23] Liu R , Qian K , He X , Li H . Integration of scRNA‐seq data by disentangled representation learning with condition domain adaptation. BMC Bioinf. 2024;25(1):116. 10.1186/s12859-024-05706-9 PMC10944609 38493095

[24] Gayoso A , Steier Z , Lopez R , Regier J , Nazor KL , Streets A , et al. Joint probabilistic modeling of single‐cell multi‐omic data with totalVI. Nat Methods. 2021;18(3):272–282. 10.1038/s41592-020-01050-x PMC7954949 33589839

[25] Gong B , Zhou Y , Purdom E . Cobolt: integrative analysis of multimodal single‐cell sequencing data. Genome Biol. 2021;22(1):1–21. 10.1186/s13059-021-02556-z PMC8715620 34963480

[26] Zuo C , Dai H , Chen L . Deep cross‐omics cycle attention model for joint analysis of single‐cell multi‐omics data. Bioinformatics. 2021;37(22):4091–4099. 10.1093/bioinformatics/btab403 34028557

[27] Zhan Y , Liu J , Ou‐Yang L . scMIC: a deep multi‐level information fusion framework for clustering single‐cell multi‐omics data. IEEE J Biomed Health Inform. 2023;27(12):6121–6132. 10.1109/JBHI.2023.3317272 37725723

[28] Hu D , Liang K , Dong Z , Wang J , Zhao Y , He K . Effective multi‐modal clustering method via skip aggregation network for parallel scRNA‐seq and scATAC‐seq data. Briefings Bioinf. 2024;25(2):bbae102. 10.1093/bib/bbae102 PMC10944573 38493338

[29] Zhang T , Zhang H , Ren J , Wu Z , Zhao Z , Wang G . scDRMAE: integrating masked autoencoder with residual attention networks to leverage omics feature dependencies for accurate cell clustering. Bioinformatics. 2024;40(10):btae599. 10.1093/bioinformatics/btae599 PMC11513018 39404795

[30] He K , Zhang X , Ren S , Sun J . Deep residual learning for image recognition. In: Proceedings of the IEEE/CVF Conference on Computer Vision and Pattern Recognition. Piscataway, NJ: IEEE; 2016. p. 770–778.

[31] Chen H , Ryu J , Vinyard ME , Lerer A , Pinello L . SIMBA: single‐cell embedding along with features. Nat Methods. 2024;21(6):1003–1013. 10.1038/s41592-023-01899-8 PMC11166568 37248389

[32] Li T , Qian K , Wang X , Li WV , Li H . scBiG for representation learning of single‐cell gene expression data based on bipartite graph embedding. NAR Genom Bioinform. 2024;6(1):lqae004. 10.1093/nargab/lqae004 PMC10823585 38288376

[33] Zhang X , Qian K , Li H . Structure‐preserved integration of scRNA‐seq data using heterogeneous graph neural network. Briefings Bioinf. 2024;25(6):bbae538. 10.1093/bib/bbae538 PMC11500609 39446194

[34] Meilă M . Comparing clusterings—An information based distance. J Multivariate Anal. 2007;98(5):873–895.

[35] Knops ZF , Maintz JA , Viergever MA , Pluim JP . Normalized mutual information based registration using *k*‐means clustering and shading correction. Med Image Anal. 2006;10(3):432–439. 10.1016/j.media.2005.03.009 16111913

[36] Cai D , He X , Han J . Document clustering using locality preserving indexing. IEEE Trans Knowl Data Eng. 2005;17(12):1624–1637.

[37] McInnes L , Healy J , Melville J . UMAP: uniform manifold approximation and projection for dimension reduction. arXiv. 2018.

[38] He X , Deng K , Wang X , Li Y , Zhang Y , Wang M . LightGCN: simplifying and powering graph convolution network for recommendation. In: Proceedings of the 43rd International ACM SIGIR Conference on Research and Development in Information Retrieval. New York: ACM; 2020. p. 639–648.

[39] Gao H , Xu Y , Yin Y , Zhang W , Li R , Wang X . Context‐aware QoS prediction with neural collaborative filtering for internet‐of‐things services. IEEE Internet Things J. 2019;7(5):4532–4542.

[40] Wolf FA , Angerer P , Theis FJ . SCANPY: large‐scale single‐cell gene expression data analysis. Genome Biol. 2018;19:1–5. 10.1186/s13059-017-1382-0 PMC5802054 29409532

[41] Lin X , Tian T , Wei Z , Hakonarson H . Clustering of single‐cell multi‐omics data with a multimodal deep learning method. Nat Commun. 2022;13(1):7705. 10.1038/s41467-022-35031-9 PMC9748135 36513636

[42] Luo Z , Xu C , Zhang Z , Jin W . A topology‐preserving dimensionality reduction method for single‐cell RNA‐seq data using graph autoencoder. Sci Rep. 2021;11(1):20028. 10.1038/s41598-021-99003-7 PMC8501122 34625592

[43] Ray‐Jones H , Sung CK , Chan LT , Haglund A , Artemov P , Della Rosa M , et al. Genetic coupling of enhancer activity and connectivity in gene expression control. Nat Commun. 2025;16(1):970. 10.1038/s41467-025-55900-3 PMC11772589 39870618

[44] Schmidt F , Kern F , Schulz MH . Integrative prediction of gene expression with chromatin accessibility and conformation data. Epigenetics Chromatin. 2020;13(1):4. 10.1186/s13072-020-0327-0 PMC7003490 32029002

[45] Reed TJ , Tyl MD , Tadych A , Troyanskaya OG , Cristea IM . Tapioca: a platform for predicting de novo protein–protein interactions in dynamic contexts. Nat Methods. 2024;21(3):488–500. 10.1038/s41592-024-02179-9 PMC11249048 38361019

[46] Gao Z , Jiang C , Zhang J , Jiang X , Li L , Zhao P , et al. Hierarchical graph learning for protein–protein interaction. Nat Commun. 2023;14(1):1093. 10.1038/s41467-023-36736-1 PMC9968329 36841846

[47] Zhang X , Lan Y , Xu J , Quan F , Zhao E , Deng C , et al. CellMarker: a manually curated resource of cell markers in human and mouse. Nucleic Acids Res. 2019;47(D1):D721–D728. 10.1093/nar/gky900 PMC6323899 30289549

[48] Cusanovich DA , Daza R , Adey A , Pliner HA , Christiansen L , Gunderson KL , et al. Multiplex single‐cell profiling of chromatin accessibility by combinatorial cellular indexing. Science. 2015;348(6237):9–914. 10.1126/science.aab1601 PMC4836442 25953818

[49] Hofmann T . Probabilistic latent semantic indexing. In: Proceedings of the 22nd Annual International ACM SIGIR Conference on Research and Development in Information Retrieval. New York: ACM; 1999. p. 50–57.

[50] Pedregosa F , Varoquaux G , Gramfort A , Michel V , Thirion B , Grisel O , et al. Scikit‐learn: machine learning in python. J Mach Learn Res. 2011;12:2825–2830.

[51] Kingma DP , Ba J . Adam: a method for stochastic optimization. arXiv. 2014.

[52] Blondel VD , Guillaume JL , Lambiotte R , Lefebvre E . Fast unfolding of communities in large networks. J. Stat Mech. 2008;2008(10):P10008.

[53] Ma S , Zhang B , LaFave LM , Earl AS , Chiang Z , Hu Y , et al. Chromatin potential identified by shared single‐cell profiling of RNA and chromatin. Cell. 2020;183(4):1103–1116. 10.1016/j.cell.2020.09.056 PMC7669735 33098772

[54] Hu Y , Wan S , Luo Y , Li Y , Wu T , Deng W , et al. Benchmarking algorithms for single‐cell multi‐omics prediction and integration. Nat Methods. 2024;21(11):2182–2194. 10.1038/s41592-024-02429-w 39322753

[55] Chen S , Lake BB , Zhang K . High‐throughput sequencing of the transcriptome and chromatin accessibility in the same cell. Nat Biotechnol. 2019;37(12):1452–1457. 10.1038/s41587-019-0290-0 PMC6893138 31611697

