# Supplementary material for: scMOG: A graph neural network method for regulatory relationship‐preserving single‐cell multi‐omics integration
Source: Quant Biol. 2026 May 6;14(3):e70041. doi: 10.1002/qub2.70041 (PMC13642094; doi:10.1002/qub2.70041)
Supplement: Supplementary file 1 [file QUB2-14-e70041-s001.docx]

**Supplementary Materials for “scMOG: A graph neural network method for regulatory relationship-preserving single-cell multi-omics integration”**

**Yucheng Lu, Xun Zhang, Hongwei Li**

**November 5, 2025**

**Supplementary Tables**

Table S1. Introduction of nine comparison methods.

Table S2. Clustering performance using scMOG’s two omics-specific embeddings.

Table S3. Preprocessing Steps and parameter settings of nine comparison methods.

**Supplementary Notes**

Supplementary Note 1. Introduction of clustering evaluation metrics ACC, NMI, and ARI.

Supplementary Note 2. Calculation of MSE and Accuracy.

**Supplementary Figures**

Figure S1. Comprehensive comparison of different algorithms on mouse skin dataset Ma-1.

Figure S2. Comprehensive comparison of different algorithms on mouse skin dataset Ma-2.

Figure S3. Comprehensive comparison of different algorithms on human PBMC dataset PBMC-1.

Figure S4. Comprehensive comparison of different algorithms on human PBMC dataset PBMC-2.

Figure S5. Comprehensive comparison of different algorithms on mouse spleen and lymph node dataset SLN208-1.

Figure S6. Comprehensive comparison of different algorithms on mouse spleen and lymph node dataset SLN208-2.

Figure S7: UMAP visualization illustrating improved cross-omics mixing after scMOG integration.

Figure S8. MSE values computed by different methods on PBMC-1 and PBMC-2 datasets.

Figure S9. Visualization of differentially expressed genes in the PBMC-2 dataset.

Figure S10. Line graphs of ACC, NMI, and ARI values of scMOG under different parameter settings.

**References**

1. **Supplementary Tables**

**Table S1:** Introduction of nine comparison methods.

| Methods | Version | Software | Downloads URLS |
| --- | --- | --- | --- |
| scMDC | 1.0.1 | Python-3.9.17 | GitHub website (xianglin226/scMDC)  ) |
| scMIC | NA | Python-3.9.17 | GitHub website (Zyl-SZU/scMIC) |
| DCCA | 0.0.1 | Python-3.9.17 | GitHub website (cmzuo11/DCCA) |
| Cobolt | 1.0.1 | Python-3.8.19 | GitHub website (epurdom/cobolt) |
| TotalVI | 0.3 | Python-3.8.16 | GitHub website (YosefLab/totalVI_reproducibility) |
| PCA | NA | Python-3.9.17 | NA |
| scGAE | NA | Python-3.7.15 | GitHub website (ZixiangLuo1161/scGAE) |
| SIMBA | 1.1 | Python-3.9.12 | GitHub website (pinellolab/simba) |
| scDRMAE | NA | Python-3.9.17 | GitHub website (hongfeiZhang-source/scDRMAE) |

**Table S2:** Clustering performance using scMOG’s two omics-specific embeddings.

| Dataset | Embedding from encoder 1 | | | Embedding from encoder 2 | | |
| --- | --- | --- | --- | --- | --- | --- |
|  | ACC | NMI | ARI | ACC | NMI | ARI |
| Ma-1 | 0.7413 | 0.7777 | 0.6398 | 0.6521 | 0.7155 | 0.5354 |
| Ma-2 | 0.605 | 0.6682 | 0.4904 | 0.5205 | 0.6152 | 0.4135 |
| PBMC-1 | 0.5606 | 0.7206 | 0.5127 | 0.5513 | 0.7217 | 0.4953 |
| PBMC-2 | 0.6822 | 0.737 | 0.6259 | 0.7435 | 0.7493 | 0.7487 |
| Chen | 0.8069 | 0.725 | 0.7061 | 0.6041 | 0.4864 | 0.4506 |
| Spector | 0.6912 | 0.7115 | 0.579 | 0.5622 | 0.6604 | 0.4746 |
| SLN208-1 | 0.6348 | 0.6797 | 0.5867 | 0.5107 | 0.6032 | 0.4514 |
| SLN208-2 | 0.6516 | 0.6552 | 0.5986 | 0.4639 | 0.5744 | 0.4185 |

**Table S3:** Preprocessing steps and parameter settings of nine comparison methods.

| Methods | Data preprocessing | Highly variable genes required | Parameters |
| --- | --- | --- | --- |
| scMDC | 1. Selection of highly variable features. 2. Normalization. each cell’s count is normalized by a size factor, which is calculated as the library size of each cell divided by the median library size of all cells. Then, a log transformation is applied, followed by scaling to unit variance while adjusting to zero mean. | 2000  (default) | Default |
| scMIC | 1. For the gene expression matrix: First, highly variable genes are selected. Then, normalization is performed based on the library size of each cell. A natural logarithm transformation ln(x+1) is applied, followed by Z-score normalization to ensure all genes have a mean of 0 and variance of 1. Finally, PCA is performed to extract the top 100 principal components. 2. For the chromatin accessibility matrix: LSI (latent semantic indexing) analysis is applied. 3. For the protein matrix: The same normalization method as the gene expression matrix is used. Then, the normalized cells are further scaled to have a mean of 0 and variance of 1. | 3000  (default) | Default |
| DCCA | 1. For the gene expression data: First, filtering of cells and genes is performed. Then, a log transformation is applied. Finally, the top 2000 highly variable genes are selected. 2. For the chromatin accessibility matrix: First, filtering of cells and peaks is performed. Then, the top 10,000 highly variable peaks are selected. | 2000  (default) | Default |
| Cobolt | 1. Quality control. Features with total counts lower than the 70th percentile or higher than the 99th percentile are removed. | No | Default |
| TotalVI | 1. Quality control. Filtering is performed on both cells and features. 2. Highly variable genes are selected. | 4000  (default) | Default |
| PCA | 1. Quality control. Genes expressed in fewer than three cells are first filtered. 2. A log transformation is applied using the natural logarithm function ln(x+1). 3. PCA is performed, and the top 64 principal components are selected. | No | Default |
| scGAE | 1. Quality control. Genes expressed in fewer than 3 cells and cells expressing fewer than 200 genes are filtered. 2. Normalization: each cell is divided by the total gene count and then multiplied by 10,000. 3. Log transformation: data is processed using the ln(x+1) function. 4. Scaling: Each gene’s expression level is scaled to have a mean of 0 and variance of 1. | No | Default |
| SIMBA | 1. For the gene expression matrix: quality control is performed first, removing genes expressed in fewer than 3 cells. Normalization is then applied based on library size. Finally, a log transformation is performed. 2. For the chromatin accessibility and protein matrices: Quality control is performed by removing features present in fewer than three cells. | No | Default |
| scDRMAE | 1. Normalization: each cell is divided by the total count of the corresponding feature and then multiplied by 10,000. 2. Log transformation: data is processed using the ln(x+1) function. 3. Selection of highly variable features. | 3000  (default) | Default |

2. **Supplementary Notes**

**Supplementary Note 1:** Clustering evaluation metrics

**ACC^[1]^:** ACC is a commonly used metric for evaluating clustering results, measuring the degree of alignment between clustering labels and ground truth labels. Accuracy represents the proportion of correctly clustered samples to the total number of samples. During computation, the Hungarian algorithm is first applied to align the clustering assignments with cell type labels, and then ACC is calculated. The value ranges from 0 to 1, where a value closer to 1 indicates better clustering performance.

**NMI^[2]^:** NMI measures the amount of shared information between two clustering results. It is derived from mutual information, which quantifies the dependency between two random variables. NMI normalizes mutual information, eliminating the influence of the number of clusters, making it suitable for datasets of different scales. The value ranges from 0 to 1, where a value closer to 1 indicates better clustering performance.

**ARI^[3]^:** ARI quantifies the similarity between two clustering results while considering the impact of randomness in clustering evaluation. It is an adjusted version of the Rand Index that accounts for chance grouping, avoiding biases caused by differences in sample sizes or random assignments. The value ranges from -1 to 1, where a value closer to 1 indicates better clustering performance.

**Supplementary Note 2:** Calculation methods of MSE and Accuracy values

**MSE**: MSE is used to measure the error between the similarity matrix computed from low-dimensional feature embeddings and the feature association matrix. A lower MSE value indicates that the low-dimensional embeddings retain more of the true regulatory information, whereas a higher MSE suggests that the feature embeddings capture less biological information. The calculation method is shown below:

$$\mathrm{MSE}=\frac{1}{m_{1}\times m_{2}}\left\| \mathrm{Sigmoid}\left( \frac{\mathbf{E}_{f_{1}}\cdot\mathbf{E}_{f_{2}}^{T}}{\left\| \mathbf{E}_{f_{1}} \right\|_{2}\cdot\left\| \mathbf{E}_{f_{2}} \right\|_{2}} \right)-\mathbf{A}_{f} \right\|_{2}^{2},$$

where $\mathbf{E}_{f_{1}}\in\mathbb{R}^{m_{1}\times d}$and $\mathbf{E}_{f_{2}}\in\mathbb{R}^{m_{2}\times d}$ represent the low-dimensional embeddings of genes and peaks, respectively, and $d$ denotes the dimensi on of the feature embeddings. $\mathbf{A}_{f}$ is the gene-peak association matrix computed based on genomic annotations^[4]^, which is a binary matrix. For a fair comparison, we take the intersection of the features obtained by scMOG and SIMBA.

**Accuracy:** Accuracy is used to measure the proportion of correctly predicted similar protein pairs obtained by different methods. Euclidean distance is used to assess protein similarity, and the top 2% most similar protein pairs are selected. The proportion of correctly predicted protein pairs is then determined by querying the STRING database.

3. **Supplementary Figure**

Figure S1: Comprehensive comparison of different algorithms on the mouse skin dataset. (A) UMAP visualization of cell embeddings obtained by different algorithms, with colors indicating the ground truth cell types. (B) Comparison of ACC, NMI, and ARI scores computed by different algorithms on the mouse skin dataset. scMOG achieves the best performance in both the UMAP visualization (Figure S1A) and quantitative metrics (Figure S1B). It demonstrates well-separated cell clusters and outperforms other methods in ACC, NMI, and ARI, indicating its effectiveness in capturing the biological differences among real cell types. DCCA and PCA perform moderately well, whereas scGAE and scMDC rank lower in terms of performance.

Figure S2: Comprehensive comparison of different algorithms on the mouse skin dataset. (A) UMAP visualization of cell embeddings obtained by different algorithms, with colors indicating the ground truth cell types. (B) Comparison of ACC, NMI, and ARI scores computed by different algorithms on the mouse skin dataset. From the UMAP visualization, scMOG effectively preserves the structure of cell types, with clear boundaries between different cell clusters, demonstrating strong cell type separation ability. Compared to other methods such as PCA, Cobolt, and DCCA, scMOG exhibits more compact clustering with lower levels of cell type mixing. Among the comparison methods, scMDC and scMIC also show good cell cluster separation in the UMAP visualization. The quantitative metrics further validate the advantage of scMOG in multi-omics data integration. While its ACC and NMI scores are slightly lower than those of DCCA, scMOG achieves a higher ARI score.

Figure S3: Comprehensive comparison of different algorithms on the human PBMC dataset. (A) UMAP visualization of cell embeddings obtained by different algorithms, with colors indicating the ground truth cell types. (B) Comparison of ACC, NMI, and ARI scores computed by different algorithms on the human PBMC dataset. In Figure S3A, the UMAP visualizations of cell embeddings from different methods indicate that scMDC and scMOG achieve clearer separation of different cell populations, effectively preserving the biological heterogeneity of multi-omics data. In contrast, the traditional dimensionality reduction method PCA exhibits noticeable cell-type mixing. Figure S3B further quantifies the performance of each method in terms of ACC, NMI, and ARI. scMOG ranks in the middle for ACC and ARI scores, but achieves the second-highest NMI score.

Figure S4: Comprehensive comparison of different algorithms on the human PBMC dataset. (A) UMAP visualization of cell embeddings obtained by different algorithms, with colors indicating the ground truth cell types. (B) Comparison of ACC, NMI, and ARI scores computed by different algorithms on the human PBMC dataset. Figure S4A displays the UMAP visualization results of cell embeddings from different methods on the human PBMC-2 dataset, where different colors represent distinct cell types. It can be observed that different methods vary in their ability to separate cell types, with scMOG and scMDC exhibiting superior cell type separation. Figure S4B compares the performance of these methods in terms of ACC, NMI, and ARI. The results indicate that scMOG achieves relatively strong performance, ranking in the middle for ACC and NMI scores, but securing a top-three position in ARI score.

Figure S5: Comparative analysis of different algorithms on the mouse spleen and lymph node dataset. (A) UMAP visualization of cell embeddings obtained by different algorithms, with colors indicating the ground truth cell types. (B) Comparison of ACC, NMI, and ARI scores computed by different algorithms on the mouse spleen and lymph node dataset. This dataset comprises two modalities: transcriptomics and proteomics. scMOG demonstrates the best performance in both UMAP visualization (Figure S5A) and quantitative evaluation (Figure S5B). Figure S5A visualizes the cell embedding distributions generated by different methods using UMAP. While all methods exhibit varying degrees of cell type mixing, scMOG shows a lower level of mixing. For example, MZ B cells and Mature B cells, which are relatively abundant in the dataset, are better separated in scMOG embeddings. In terms of quantitative metrics, scMOG achieves the highest ACC, NMI, and ARI scores. Notably, in the ARI metric, scMOG significantly outperforms some benchmark methods.

Figure S6: Comparative analysis of different algorithms on the mouse spleen and lymph node dataset. (A) UMAP visualization of cell embeddings obtained by different algorithms, with colors indicating the ground truth cell types. (B) Comparison of ACC, NMI, and ARI scores computed by different algorithms on the mouse spleen and lymph node dataset. This dataset includes two modalities: transcriptomics and proteomics. scMOG achieves the best performance in both UMAP visualization (Figure S6A) and quantitative metrics (Figure S6B). The UMAP visualization in Figure S6A demonstrates noticeable differences in clustering performance among different methods. Compared to other approaches, scMOG more effectively separates cDC1s from cDC2s, yielding clearer boundaries between cell clusters. In terms of quantitative evaluation, scMOG achieves the best results in ACC, NMI, and ARI. Notably, scMOG outperforms several benchmark methods significantly in both the ARI and ACC metrics.

Figure S7: UMAP visualization illustrating improved cross-omics mixing after scMOG integration. Colors indicate omics type. Compared with the original embeddings, scMOG produces more intermixed cross-omics distributions while preserving local structures. (A) UMAP visualization of cells from transcriptomic data and epigenomic/proteomic data across four datasets (Ma-1, Ma-2, PBMC-1, PBMC-2). (B) UMAP visualization of multi-omics cells from four additional datasets (Chen, Spector, SLN208-1, SLN208-2).

Figure S8: MSE values computed by different methods on the PBMC-1 and PBMC-2 datasets. The last column represents the MSE value obtained by scMOG, where a lower value indicates that more interaction information between features has been captured.

Figure S9: Visualization of differentially expressed genes in the PBMC-2 dataset (A) Bubble plot showing the expression distribution of the top three differentially expressed genes predicted in each cluster on the PBMC-2 dataset. (B) UMAP visualization of the differentially expressed genes *A2M*, *BANK1*, and *RALGPS2* on the same dataset.

Figure S10: Line graphs of ACC, NMI, and ARI values for scMOG under different parameter settings. (A) shows the variation of ACC, NMI, and ARI values under different proportions of the contrastive learning module in the loss function. (B) presents the variation of ACC, NMI, and ARI values under different proportions of the omics alignment module in the loss function. (C) illustrates the variation of ACC, NMI, and ARI values under different clustering resolutions.

4. **References**
[1] Cai D, He X, Han J. Document clustering using locality preserving indexing[J]. IEEE Transactions on Knowledge and Data Engineering, 2005, 17(12): 1624-1637.
[2] Knops Z F, Maintz J B A, Viergever M A, et al. Normalized mutual information based registration using k-means clustering and shading correction[J]. Medical Image Analysis, 2006, 10(3): 432-439.
[3] Meilă M. Comparing clusterings—an information based distance[J]. Journal of Multivariate Analysis, 2007, 98(5): 873-895.
[4] Chen H, Ryu J, Vinyard M E, et al. SIMBA: single-cell embedding along with features[J]. Nature Methods, 2024, 21(6): 1003-1013.
